# TO LOVE AND LOATHE: EXAMINING THE COSTS AND BENEFITS OF AMBIVALENT TIES IN OLDER ADULTHOOD

**DOI:** 10.1093/geroni/igac059.954

**Published:** 2022-12-20

**Authors:** Lucas Hamilton, Siyun Peng, Anne Krendl, Brea Perry

**Affiliations:** Indiana University, Bloomington, Indiana, United States; Indiana University, Bloomington, Indiana, United States; Indiana University, Bloomington, Indiana, United States; Indiana University, Bloomington, Indiana, United States

## Abstract

Ambivalent ties are relationships that offer support but beget stress, which generally has a detrimental impact on health. Existing theory suggests that older adults gradually remove such ties over time; however, it is not uncommon for ambivalence to exist in older adults’ close relationships (i.e., partners, children). Social network data was used from 286 older adults with about half having mild cognitive impairment. Roughly two-thirds of the sample reported at least one ambivalent tie, most commonly partners, children, and friends. Logistic regressions revealed distinct characteristics of these ties. Participants who reported at least one ambivalent tie (most notably, partners and friends) had social networks with structures known to confer cognitive benefits. Importantly, these effects dissipate with diminished cognitive status. Altogether, ambivalent ties may confer benefits when resources are available to manage such relationships. When resources are taxed, however, ambivalent ties may contribute to cascading health declines.

